# Twenty Years' Progress of a Great Charity

**Published:** 1917-07-14

**Authors:** 


### GUY'S HOSPITAL.

### Twenty Years' Progress of a Great Charity.

The publication of the annual report of Guy's Hospital reminds us of the turning-point in the history of the great South London Hospital, in the year 1896, which led to the steady progress of the twenty years completed by the twelve months' work of which the report is the record. It will be remembered that the annual revenue of the hospital for a long time prior to 1896 had been insufficient to meet an expenditure less than half that of the present day. This was so because the capital, under the terms of the founder's will, had all been invested in land which in later years seriously depreciated in value. Approximately the income was £20,000 and the expenditure £40,000, and to meet this alarming situation 100 beds had been put out of use, and the Governors were seriously considering the closing of more wards.

At this crisis H.R.H. the Prince of Wales was approached and asked to take the chair at a festival dinner in view of the serious state of the finances of Guy's Hospital. H.R.H. did not see his way to accept the invitation, and shortly afterwards the then President, the first Lord Aldenham, who was Governor of the Bank of England, had a conference with Mr. Henry C. Burdett, in the course of which the whole position of Guy's Hospital was carefully gone into. The needs and changes to command success were fully discussed and a course agreed to. The plan included the holding of a great banquet at the Imperial Institute, the retirement of the President and the Treasurer, and the appointment of the best men possible to succeed Lord Aldenham and Mr. Edward Lushington. In the result everybody connected with Guy's Hospital combined to support warmly the new scheme, and to give personal service to ensure its success. One of the most noticeable features was the effort made by the medical staff, who organised a far-reaching scheme for the raising of money by their efforts and influence, which resulted in a great sum.

Everybody worked with a will, and by June 10, 1896, the date of the festival banquet at the Imperial Institute, a record sum had been raised. That sum amounted to £170,000, plus £3,300 in New Annual Subscriptions. The Prince of Wales (King Edward VII.) was again approached, and, in the altered circumstances, consented to preside at the Imperial Institute banquet. H.R.H. took a great personal interest in everything connected with it, agreed to succeed Lord Aldenham as President of Guy's Hospital, and invited one of the Governors, Mr. H. Cosmo Bonsor, to a Conference, as he felt him to be the best man in the circumstances to succeed Mr. Lushington as Treasurer. Everybody knows that Mr. Cosmo Bonsor proved himself to be an ideal Treasurer, justifying the Prince's opinion, and that there is probably no man who has held a similar office in the last quarter of a century in connection with one of our great hospitals who has rendered anything like the same service, or attained to anything like equal results and success with those achieved by Mr. Cosmo Bonsor, who is now President of Guy's Hospital, the treasurership being held by Viscount Goschen.

The banquet of June 10 had novel features; it was, we believe, the first of its kind where the guests were seated in parties at small tables. The largest table was that at which the Prince of Wales presided, and he had round him a brilliant company, including the Right Hon. A. J. Balfour, Lord Roberts, Dr. Page-Smith, Sir Samuel Wilks, and the two organisers of the festival, Sir Alfred Fripp and Sir Cooper Perry, as well as other distinguished men interested in the hospital. After the dinner a reception was held, at which between six and seven thousand people were present.

The period will be fixed when we narrate that one of the features of the festival was a display of motor cars, the industry being then in its infancy and the Act which required a man with a red flag to walk in front of every self-propelled vehicle had not yet been repealed. The guests were allowed to take rides round the quadrangle of the Institute at a charge of a sovereign a journey.

From that time to this the financial position of the hospital has gone from strength to strength, and what is equally important is that the hospital and all that belongs to it, its medical school, its nursing service, its appointments and equipments, previously starved for lack of funds, have in the same time, under careful, untiring, and skilful management, been maintained and improved until to-day the institution is a model of efficiency.

The amount aimed at for a re-endowment fund was half a million; up to December 31, 1916, £361,600 had been received, and the total of dividends last year from this source was £13,600. This additional and secured income is especially valuable just now, for Guy's has been hit by the war not less than other English hospitals. The strain, as the Treasurer's report states, is felt in two directions—the shortage of staff and the cost of supplies. In respect of the first factor, had it not been for the devotion of those members of the staff who are not serving in the war a serious contraction of the work of the hospital would before this have been inevitable. Before the war there were fifty members of the permanent and twenty of the resident staff, and at the present time the corresponding numbers are twenty-eight and seven.

Guy's has co-operated with the military authorities in two principal ways. For the use of wounded officers forty-six cubicles have been provided, which were used by 362 patients in 1916. The other chief help has been afforded by the setting apart of Evelyn Ward for the reception of recruits who by treatment could be converted from unfit to serviceable soldiers.

A list published recently by the *Guy's Hospital Gazette* (to which we are indebted for some of the information contained in the opening paragraphs of this article) gave particulars of nearly 500 nurses trained at Guy's who are on war service, in which a number of them have gained distinctions. A heavy loss to the nursing strength of the hospital, mentioned in the report, was the resignation of the matron, Miss Haughton, following on her severe illness.

As one instance of the increased cost of maintenance during the last few years may be mentioned coal. The average price per ton rose from 20s. in 1913 to 36s. 5d. in 1916, and the gross cost from £4,993 to £10,728. The consumption is necessarily large, as the lighting of the hospital by electricity, the laundry, lifts, and other mechanical power, as well as heating, are provided from a central station.

The progressive character of the administration is shown in many ways, and one of them is the provision of private wards for patients who cannot afford the charges of a nursing home. A certain number of these wards are available at a fee of £3 10s. a week, which covers the cost of nursing and maintenance. The patients are under the care of the house officers, but should they require the attendance of one of the hospital physicians or surgeons they must make their own arrangements as to fees.

The financial position of the hospital at the end of 1916 was very sound. The total income (including legacies amounting to £27,581) was £103,469, and the total expenditure £89,943. But it must be noted that there was no extraordinary expenditure on new buildings and equipment, the sound policy of the hospital in respect of renovations and extensions having necessarily been modified to meet existing circumstances.

From a glance at the statistical tables it is learned that although the daily average bed occupancy in 1916 was slightly higher than in 1915, a smaller number of in-patients were treated to a conclusion, the reason being that the average stay was increased. The total out-patients fell from 110,932 to 106,326, and the attendances from 457,877 to 428,944. The statistics of the laundry record that 1,346,920 pieces were dealt with in the twelve months.

These figures illustrate in a forcible way the extent of the work of Guy's Hospital in a single year.

